# Genetic Diversity and Environmental Influence on Yield and Yield-Related Traits of Adzuki Bean (*Vigna angularis* L.)

**DOI:** 10.3390/plants11091132

**Published:** 2022-04-21

**Authors:** Liangliang Hu, Gaoling Luo, Xu Zhu, Suhua Wang, Lixia Wang, Xuzhen Cheng, Honglin Chen

**Affiliations:** 1 Institute of Crop Sciences, Chinese Academy of Agricultural Sciences, Beijing 100081, China; hu15101081634@163.com (L.H.); wangsuhua@caas.cn (S.W.); wanglixia03@caas.cn (L.W.); 2 Institute of Rice Research, Guangxi Academy of Agricultural Sciences, Nanning 530007, China; luogaoling@163.com; 3 Nanyang Academy of Agricultural Science, Nanyang 473000, China; zhuxu315@126.com

**Keywords:** adzuki bean (*Vigna angularis* L.), germplasm, genetic variability, yield-related traits, domestication

## Abstract

Adzuki beans are an important food legume crop in East Asia. A large number of adzuki bean accessions are maintained in the Chinese national seed genebank. A collection of 59 elite cultivars, 389 landraces, and 27 wild adzuki beans were selected and phenotyped extensively for yield and yield-related traits at two different locations (Nanning and Nanyang, China). Ten agronomic and yield-related traits were scored, and the data were subjected to analysis of variance (ANOVA), principal component analysis (PCA), correlation, and cluster analysis. Significant variation was observed for genotypes, locations, and genotype x environment interaction for most traits. Also, there were significant differences in the phenotypes among accessions of different germplasm types. The broad-sense heritability of traits studied ranged from 4.4% to 77.8%. The number of seeds per pod (77.8%), 100-seed weight (68.0%), and number of plant branches (63.9%) had a high heritability. A total of 10 traits were transformed into 3 comprehensive factors by principal component analysis, and the first three principal component factors contributed 72.31% of the total variability. Cluster analysis categorized the 475 adzuki bean accessions into five distinct groups. The results described in this study will be useful for adzuki bean breeders for the development of varieties with high end-use quality.

## 1. Introduction

The adzuki bean (*Vigna angularis* L.) is a diploid (2*n* = 22), self-pollinating, and annual legume which belongs to the *Vigna* of *Leguminosae* [1,2]. Adzuki beans originated in China and were domesticated ~12,000 y ago, and the main growing areas were in north and northeast China and the middle and lower reaches of the Yellow River and the Yangtze River region of China [2,3]. The adzuki bean is planted in more than 30 countries in the world (although mainly in China, Japan and South Korea) [4], and accounts for planting area of nearly 0.70 million ha annually. Therefore, the adzuki bean is also known as “Asian legume crop” [5]. Adzuki beans are rich in proteins, vitamins, flavonoids, minerals, but have low calories and fat [6,7]. In addition, adzuki beans as a kind of medicinal food have antioxidant, anti-hypertensive, anti-inflammatory, anti-diabetic, and other effects [7,8,9].

Domestication has triggered a wide range of morphological and physiological traits that distinguish domesticated crops from their wild ancestors. The domestication of cultivated adzuki beans from their wild ancestors represents one of the most important events of human history [10]. The cultivated adzuki bean varieties are the result of extensive breeding and artificial selection [11]. The cultivated adzuki bean (*V. angularis* var. *angularis*) differs from the wild adzuki bean (*V. angularis* var. *nipponensis*) in many traits, e.g., the cultivated adzuki bean forms flowers earlier and has larger seeds than the wild adzuki bean, the seed size has markedly increased, the growth habit has changed from twining to erect stems, the plant height decreased, and the maturation period has shortened significantly. These changes contributed to an increase in yield over the adzuki bean landrace and cultivated varieties. Landraces represent an intermediate stage of domestication between the wild ancestor and modern cultivated varieties, and they are a valuable source of genetic diversity. Hence, understanding and utilizing the genetic variation in adzuki bean accessions is essential for improving the crop [12]. The yield of adzuki beans can be divided into several components. The number of plants per unit area, nodes per plant, pods per node, and seed size are the main parameters which are determined at different stages of reproductive growth. Seed number per unit land area is the most important yield component, and there is a differential response of yield components to changes in environmental conditions. Agronomic traits and yield characters of crops are mostly quantitative traits which are influenced by gene effect and environmental effect [13,14,15]. Genotype x environment interactions lead to different responses in different traits of crops, which plays an important role in the selection processes of crops. The crop yield was a comprehensive trait, and yield characters included many traits such as 100-seed weight and seed weight per plant. As a short-day crop, the yielding ability of the adzuki bean is affected by many factors such as soil fertility [16], water conditions [17], and photoperiod responses [18,19]. Environmental factors mainly influence plant physiology, especially compensatory relationships among the number of pods per plant, number of seeds per pod, and seed size.

The identification and evaluation of agronomic traits of germplasm resources is the basis of crop breeding. The methods used commonly include principal component analysis [20,21,22], cluster analysis [22,23,24], etc. In this study, we conducted field trials in two locations with a total number of 475 accessions of adzuki bean, and 10 agronomic and yield-related traits were identified. Then, the traits studied of these germplasm resources were comprehensively evaluated by correlation analysis, principal component analysis, and cluster analysis. This study aims to provide a theoretical basis for the collection, preservation, and germplasm innovation of the adzuki bean.

## 2. Results

### 2.1. Phenotypic Analysis of Traits in Different Environments

A large number of variations were observed in the 10 traits among the accessions evaluated in two locations (Table 1 and Supplementary Materials Table S1). The plant height (PH), number of pods per plant (NPP), and plot yield (PY) were among the traits with the largest ranges. PH in germplasm ranged from 13.5 cm to 130.5 cm with a mean 27.7 cm in Nanning while ranged from 30.0 cm to 193.9 cm with a mean 69.3 cm in Nanyang. NPP in germplasm ranged from 2.3 to 80.3 with a mean 15.2 in Nanning while ranged from 2.3 to 109.7 with a mean 20.2 in Nanyang. PY in germplasm ranged from 14.7 g to 1799.6 g with a mean 403.6 g in Nanning while ranged from 51.3 g to 2312.6 g with a mean 620.5 g in Nanyang. PH, nodes number of the main stem (NNS), number of plant branches (NPB), days to flowering (DF), days to maturity (DM), NPP, seeds weight per plant (SWP), and PY in Nanning were significantly higher than those in Nanyang (*p* < 0.05). Number of seeds per pod (NSP) and 100-seed weight (100-SW) had no significant difference between the two locations. NPB, PY, NPP, and SWP had a wide variation in both two locations (CV > 50%), while DM, DF, and NNS possessed a narrow variation in two locations (CV < 20%). NSP, PY, and NNS had the highest Shannon’s diversity index (SHDI) in Nanning, while the DF and NSP had the highest SHDI in Nanyang. A high level of variability and diversity exists among genotypes and locations in response to the traits scored, indicating that traits studied were influenced by genotype x environment interactions. In this study, NSP and 100-SW were less affected by the environment (since these two traits were had no significant differences in two locations).

Analysis of variance (ANOVA) (Table 2) showed that a significant block effect was observed for all traits (*p* < 0.001) studied except NSP. The genotypes (G) varied significantly in PH (*p* < 0.001), NNS (*p* < 0.001), NPB (*p* < 0.001), NSP (*p* < 0.001), 100-SW (*p* < 0.001), PY (*p* < 0.01), and DF (*p* < 0.05), but had no significant difference in DM, NPP, and SWP. Significant differences (*p* < 0.001) were recorded among the environments (E) for all the traits measured except NSP and 100-SW. There was also a high significant effect of genotype × environment (G × E) interaction on all the traits (*p* < 0.001).

### 2.2. Relationships between Latitude of Collection Site and Phenotypic Traits

**Figure 1:**
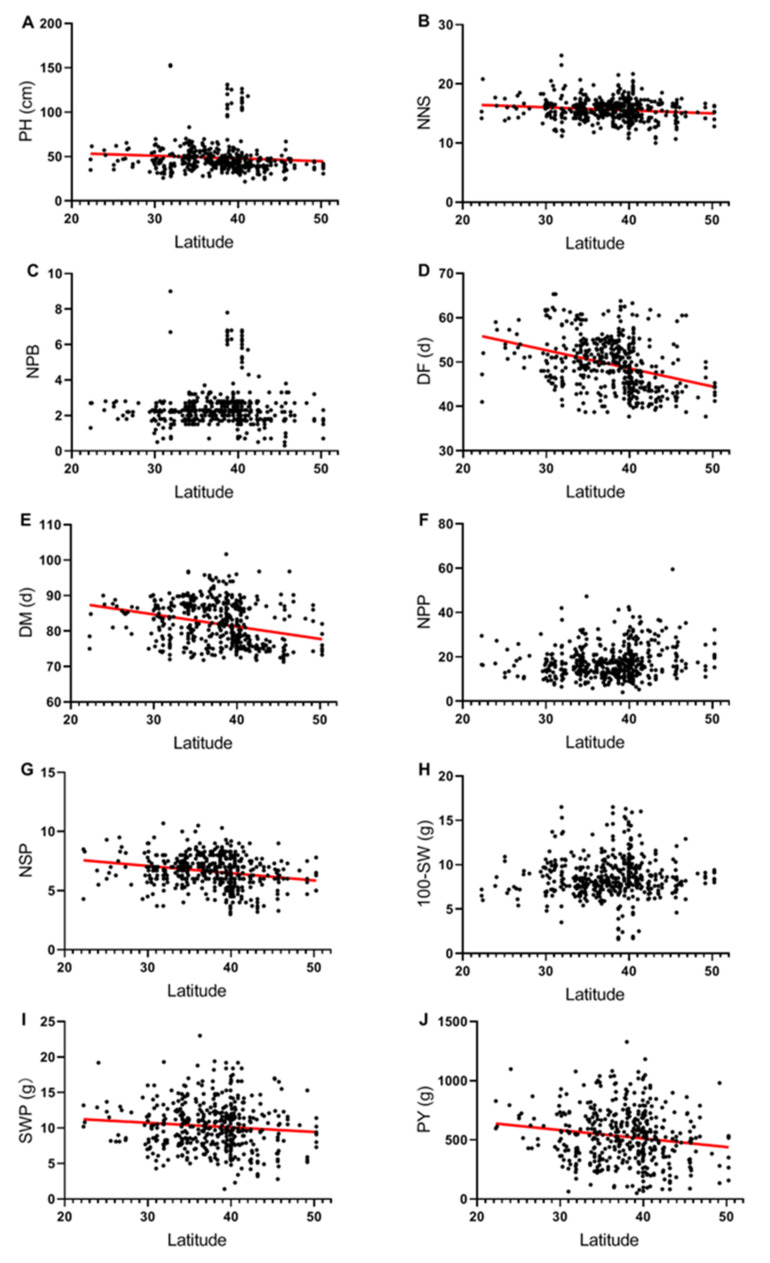
Relationship between latitude of the collection site and plant height (**A**), nodes number of the main stem (**B**), number of plant branches (**C**), days to flowering (**D**), days to maturity (**E**), number of pods per plant (**F**), number of seeds per pod (**G**), 100-seed weight (**H**), seeds weight per plant (**I**), and plot yield (**J**). Regression lines were drawn when there was a significant effect (*p* < 0.05).

For a better understanding of how the latitude of the collection site is related to agronomic and yield traits, scatter plot and linear regression analyses were conducted (Figure 1). PH, NNS, DF, DM, NSP, SWP, and PY were significantly decreased with the increasing latitude of the collection site, while NPB, NPP, and 100-SW had no significant effect by the variety of latitude. The slopes of the regression lines between latitude and traits were different. The slopes of the regression lines between latitude and DF, DM were steeper than the other traits, indicating that DF and DM were more likely to decrease with the increasing latitude of the collection site.

Linear regression analyses between DF and latitude of the collection site suggested that flowering date was delayed with increasing latitude, resulting in reproductive growth delayed and yield characters decreased. These findings indicated that accessions collected at lower latitudes have greater potential in traits related to yield, although variation is wide among accessions collected in the same latitude. The significant relationship between traits and latitude of the collection site suggests that accessions collected from different geographical latitudes are phenotypically diverse, which can be used for breeding new adzuki bean varieties adapting to various latitudinal locations.

### 2.3. Phenotypic Analysis of Traits of Different Germplasm Types

**Figure 2:**
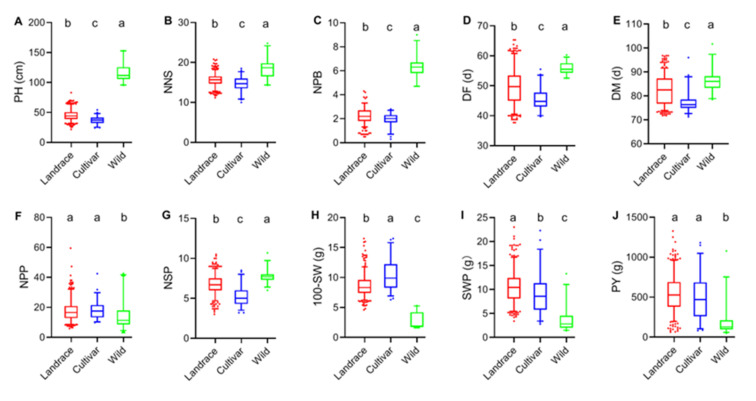
Box and bar plots of plant height (**A**), nodes number of the main stem (**B**), number of plant branches (**C**), days to flowering (**D**), days to maturity (**E**), number of pods per plant (**F**), number of seeds per pod (**G**), 100-seed weight (**H**), seeds weight per plant (**I**), and plot yield (**J**) grouped by species, in each box and bar plot the respective groups were compared using a least significant difference (LSD) test and the groups with the different letters were significantly different at alpha = 0.05. Different colors of bar represent different germplasm types. Red, blue, and green color represent landraces, cultivated varieties, and wild accessions, respectively.

A total of 475 adzuki bean accessions we selected, including 389 landraces, 59 cultivated varieties, and 27 wild accessions, and differences in phenotype were compared according to germplasm types (Figure 2). A very large variation were observed in the traits among accessions of different germplasm types. PH ranged from 95.3 cm to 153.1 cm with a mean of 115.9 cm in wild accessions, while ranged from 24.3 cm to 54.3 cm with a mean of 37.2 cm in cultivated varieties. DF ranged from 39.7 days to 55.5 days with a mean of 45.4 days in cultivated varieties, while ranged from 52.5 days to 60.3 days with a mean of 55.8 days in wild accessions. 100-SW ranged from 6.3 g to 16.5 g with a mean of 10.4 g in cultivated varieties, while ranged from 1.6 g to 5.3 g with a mean of 2.8 g in wild accessions. The other traits showed different degrees of variability in different germplasm types (*p* < 0.05).

In this study, traits related to plant growth architecture such as PH, NNS, NPB, DF, and DM were higher in wild accessions and lower in cultivated varieties. However, traits related to yield had the opposite trend except NSP. NPP, SWP, 100-SW, and PY were lower in wild accessions. 100-SW was higher in cultivated varieties, and SWP was higher in landraces, while NPP and PY had no significant differences between landraces and cultivated varieties. These findings indicated that wild accessions were more taller plants, more plant branches, longer days to flowering and maturity, while cultivated varieties were earlier maturity, larger seed size, and higher yield. This probably is the result of human selection during domestication. Furthermore, according to different breeding goals, accessions of different germplasm types can be selected for adzuki bean breeding.

### 2.4. Analysis of Genetic Components

The GCV of traits studied ranged from 2.3% to 58.7% (Table 3). The high GCV (≥20%) was observed in PH, NPB, NPP, 100-SW, and PY. The intermediate GCV (≥10% and ≤20%) was presented in NSP and SWP, while the low GCV (≤10%) in NNS, DF and DM. The PCV of traits studied ranged from 10.8% to 73.5%. PH, NPB, NPP, NSP, 100-SW, SWP, and PY had a high PCV (≥20%). However, NNS, DF, and DM showed an intermediate PCV (≥10% and ≤20%). 

Heritability is the ratio of genetic variance to the total variance (phenotypic variance). In this study, the broad-sense heritability of traits studied ranged from 4.4% to 88.3%. The NSP, 100-SW, and NPP had a high heritability (≥60%), indicating that these traits are less influenced by the environment. However, DF, DM, NPP, SWP, and PY were observed a low heritability (≤30%). Further, the genetic advance (GA) calculated had shown that the NSP, 100-SW, and NPP also possessed a high GA. These showed that NPB, NSP, and 100-SW were more affected by genes than the environment, while the DF, DM, NPP, SWP, and PY were more affected by environment.

### 2.5. Correlation Analysis of the Traits

**Figure 3:**
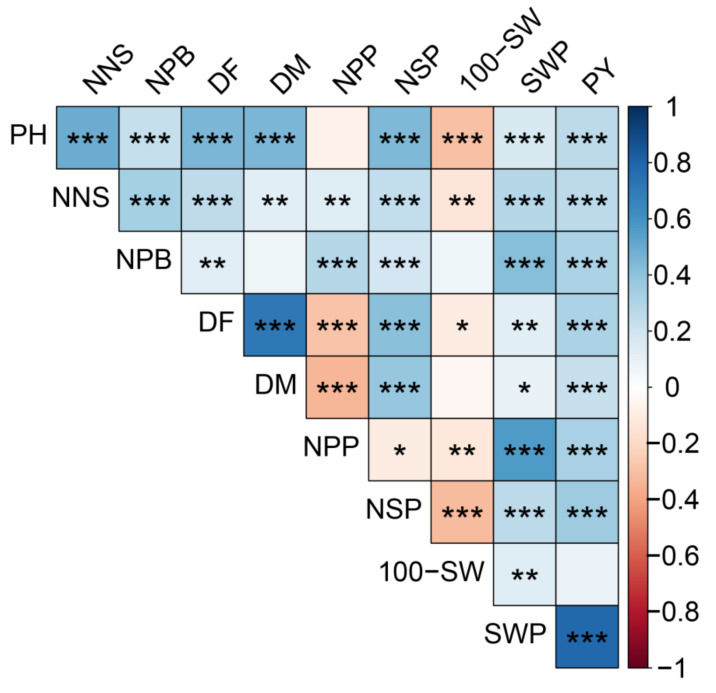
Spearman’s correlation matrix of 10 traits of 475 accessions. In the correlation matrices *, **, *** indicate significance at *p* < 0.05, *p* < 0.01 and *p* < 0.001, respectively. PH, plant height (cm); NNS, nodes number of the main stem; NPB, number of plant branches; DF, days to flowering; DM, days to maturity; NPP, number of pods per plant; NSP, number of seeds per pod; 100-SW, 100-seed weight (g); SWP, seeds weight per plant (g); PY, plot yield (g).

The correlation matrix (Figure 3) showed that PY had a positive correlation (*p* < 0.001) with SWP, PH, NNS, NPB, DF, DM, NPP, and NSP. SWP had a positive correlation (*p* < 0.05 or *p* < 0.01 or *p* < 0.001) with all other traits. However, 100-SW had a negative correlation with PH (*p* < 0.001), NNS (*p* < 0.01), DF (*p* < 0.05), NPP (*p* < 0.01), and NSP (*p* < 0.001). Further, NSP had a positive correlation (*p* < 0.001) with PH, NNS, NPB, DF, and DM, while had a negative correlation with NPP (*p* < 0.05). NPP had a positive correlation with NNS (*p* < 0.01) and NPB (*p* < 0.001), while a negative correlation with DF (*p* < 0.001) and DM (*p* < 0.001). Therefore, accessions had the taller plant height and more plant branches; they always had the higher yield, but smaller seed size. In addition, PH had a positive correlation (*p* < 0.001) with NNS, NPB, DF, and DM. DF had a positive correlation with DM (*p* < 0.001).

### 2.6. Principal Component Analysis of Traits

To reduce the redundancy of the raw data, the principal component analysis was carried out according to the 10 traits of two environments. We selected the first three principal component factors according to the principle that the eigenvalue is greater than 1, and the loading matrix and contribution to total variability to each trait were calculated (Table 4). The first three principal component factors contributed 72.31% of the total variability of the traits studied and can represent most of the variation of traits.

**Figure 4:**
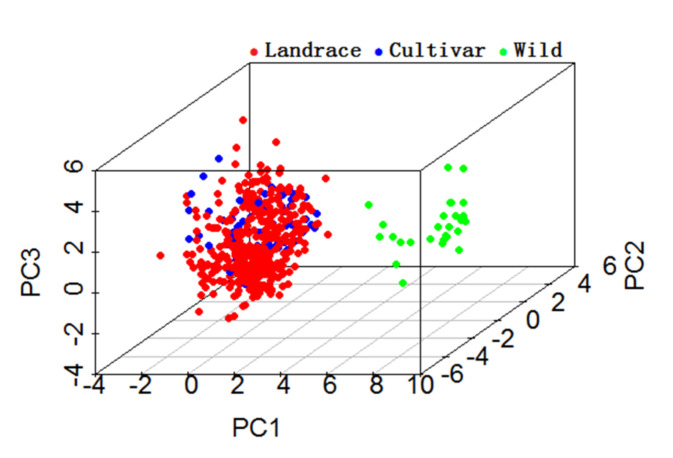
Principal component analysis (PCA) was conducted on 10 traits for the 475 accessions.

The first component (PC1) explained 31.26% of the variation and was mainly negatively correlated with PH (−0.47) and NPB (−0.41). While the second component (PC2) explained 24.49% of the variation and was more negatively correlated with DF (−0.44) and DM (−0.45). Further, the third component (PC3) explained 16.56% of the variation and was mainly correlated with NPP (0.63), 100-SW (−0.41), and SWP (0.43). In addition, the individual PCA plot (Figure 4) showed that the wild accessions can be separated from landraces and cultivated varieties by PC1, since the PC1 scores of wild accessions were greater than 0, while PC1 scores of landraces and cultivated varieties were less than 0. Landraces and cultivated varieties were dispersed by PC2, and the PC2 scores of cultivated varieties were greater than those of most landraces. These also suggested that there was wide variation in the phenotypic traits among accessions of different germplasm types.

### 2.7. Cluster Analysis of Traits

**Figure 5:**
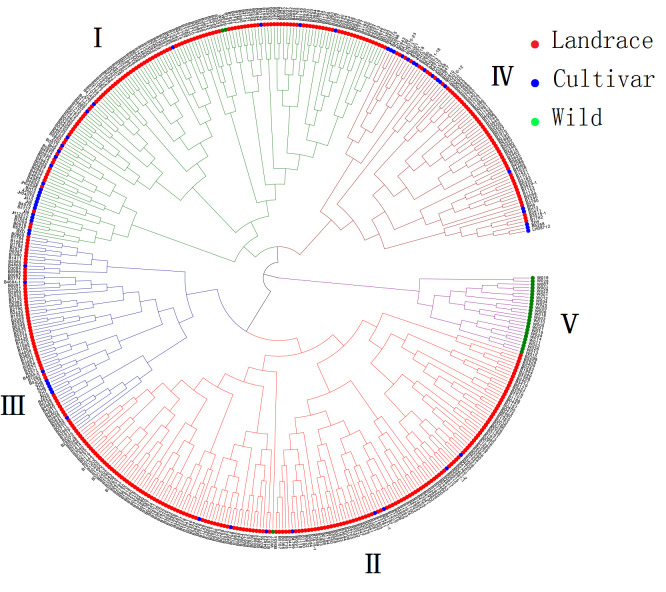
Hierarchical clustering dendrogram analysis. The 475 adzuki bean accessions were grouped into 5 groups (I–V). The clusters I, II, III, IV, and V had 143, 170, 64, 74, and 24 accessions, respectively. Dots with different color represents landraces (red), cultivated varieties (blue) and wild (green).

The 475 adzuki bean accessions were grouped into 5 groups based on the 10 traits (Figure 5 and Table 5). The clusters I, II, III, IV, and V had 143, 170, 64, 74, and 24 accessions, respectively. Cluster I included 117 landraces, 24 cultivated varieties, and 2 wild accessions. Cluster II contained 161 landraces, 8 cultivated varieties, and 1 wild accessions. Cluster III possessed 55 landraces and 9 cultivated varieties. Cluster IV was consisted of 56 landraces and 18 cultivated varieties. Cluster V had 24 wild accessions. Each group had its unique characteristics on special traits. Accessions in cluster I were shorter growth duration. On the contrary, accessions in cluster V were longer growth duration. The 100-SW, SWP, and PY were lower in both cluster I and V, and accessions in cluster V were higher in NSS and NPB. Accessions in cluster III and IV were higher 100-SW, while SWP and PY were higher in cluster III. Accessions A349, A353, A354, A355, and A357 in cluster I had earlier flowering and maturity date. Accessions A311, A447, A445, A074, and A084 in cluster IV were higher in 100-SW. Accessions A186, A348, A197, A006, and A330 in cluster III were higher SWP and higher PY. These accessions could be used for crossbreeding and germplasm innovation of adzuki bean. The clustering result was not strictly consistent with the geographic origin of the adzuki bean germplasm.

## 3. Discussion

Morphological markers can simply and intuitively display genetic polymorphisms of crop germplasm resources [25]. Identification and evaluation of agronomic traits and yield characters have important guiding significance for the classification of germplasm resources and breeding of crops [26,27]. Agronomic traits and yield characters are mostly quantitative traits, which are influenced by gene effects and environmental effects. Many crops had reported being affected by genotype x environment interactions [28,29,30,31]. Flowering is one of the most important progresses for most crops during the transition from vegetative growth to reproductive growth. Flowering time response is a quantitative trait that depends on the conditions such as photoperiod, and temperature, in the growth environment [32]. In this study, DF and DM in Nanyang were higher than those in Nanning. In addition, there were significant differences in PH, NNS, NPB, NPP, SWP, and PY in two locations, except NSP and 100-SW. Further, PH, NNS, DF, DM, NSP, SWP, and PY were significantly decreased with the increasing latitude of the collection site. 

NSP and 100-SW showed a small difference between their GCV and PCV, but other traits showed a large difference between them. The smaller the difference in value (RD) between GCV and PCV, the more genetic effects and the less environmental effects there are on the specific characters [33,34]. NSP and 100-SW possessed a high heritability and high GA, which showed that these traits were regulated by the additive genes, and with little effect from the environment. On the contrary, the low level of heritability and GA indicated traits were regulated by non-additive genes (and with more effects from the environment). Thus it is important to select traits with high GCV, heritability, and GA. Since effective selection can be achieved only when additive effect is higher than the effect from the environment [35,36].

Yield potential is thought to be partially determined by seed size, and numerous studies have tried to understand the relationship between seed size and yield in other pulses with contradictory results [37,38,39,40,41]. Cultivated cultivars exhibit significantly larger seeds than that of landraces and wild adzuki beans, which indicates that the seed size is the leading and priority component in the selection of high-yielding cultivars by breeders. In our study, PY had a positive correlation (*p* < 0.001) with SWP, PH, NNS, NPB, DF, DM, NPP, and NSP, but no significant correlation with 100-SW. A lack of correlation between seed size and yield was found in grass pea under drought conditions [37] which was consistent with our results. However, other researchers had different conclusions. A positive correlation was found between seed size and yield in chickpeas [38] and peas [39], while a negative correlation was found between seed size and yield [40]. Further, research found that seed size explained 20% of the variation in yield. However, peduncle number explained nearly 50% of the variation in yield, indicating that seed size is not the most important factor in determining seed yield [39]. In addition, a study found that crop yield within a species was more related to variations in seed number than in seed weight [41]. Hence, there appears to be no consensus regarding the relationship. We also found that there were significant differences among different germplasm types. Traits related to plant growth architecture such as PH, NNS, NPB, DF, and DM were higher in wild adzuki bean than in landraces and cultivated varieties, but yield characters were lower in wild than landraces and cultivated varieties. Similar findings have been reported [42,43]. During the domestication of the adzuki bean, the factors related to plant growth gradually decreased, while the factors related to yield gradually increased. Crop wild relatives are important resources to use as breeding materials [42]. However, the wild relatives of the adzuki bean are poorly represented in the genebank. The presumed ancestor of cultivated adzuki bean is *V. angularis* var. *nipponensis*, which is distributed in Japan, the Korean peninsula, China, Nepal, and Bhutan [11]. In comparison with wild adzuki bean, landraces and cultivated adzuki beans showed numerous differences in the traits studied, which could probably be the result of human selection during domestication. 

There are differences in the selection of methods for identification and evaluation of agronomic traits of crop germplasm resources by different researchers such as correlation analysis [44,45], principal component analysis [46], and cluster analysis [47,48]. In our study, clustering analysis showed that 475 adzuki bean accessions were distributed into five groups. The accessions in cluster I were characterized by earlier flowering and maturity dates. Accessions in cluster III were higher SWP and PY. Accessions in cluster IV were higher 100-SW. Accessions in cluster V were all belong to the wild relatives, which were taller plants, more number nodes and plant branches, longer DF and maturity, but smaller seed size. Breeders should select accessions from different clusters to maximize heterosis in their breeding work, because that similarities in accessions may result from the same accessions bearing different names due to different sources of cultivation [49]. Genetic diversity is the foundation of crop breeding work, and wide applicability has become a major aspect of crop breeding work in recent years. However, quantitative traits are controlled by many minor polygenes and are highly susceptible to environmental conditions such as photoperiod, temperature, rainfall, and other factors.

## 4. Materials and Methods

### 4.1. Plant Material

**Figure 6:**
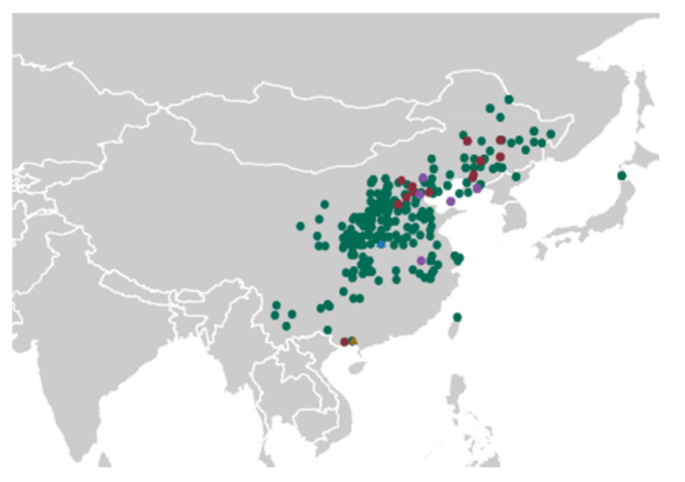
Geographical distribution of the accessions used in the study (*n* = 475). Dots with different color represents landraces (green), cultivated varieties (red) and wild (purple). Blue triangles represent the Nanyang city test sites, and brown triangles represent Nanning city test sites.

A set of 475 adzuki bean accessions representing broad genetic diversity from National Crop Genebank, Institute of Crop Science, Chinese Academy of Agriculture Science (CAAS) were selected in this field trial, including 389 landraces, 59 cultivated varieties, and 27 wild adzuki beans. A total of 469 accessions were from China, and 6 accessions were from Japan (Figure 6 and Table S1). The latitude of the collection sites ranged from 22°16′ N to 50°15′ N and longitude ranged from 100°29′ E to 140°31′ E. 

### 4.2. Experiment Location and Trial Design

The field trial was performed at two locations: Nanning city (22°13′ N, 107°45′ E) in Guangxi province and Nanyang city (34°40′ N, 112°21′ E) in Henan province in 2020. The mean daily air temperature during the experiment (June to October) was 26.9 °C and 23.0 °C in Nanning and Nanyang, respectively. Randomized complete block design was used in the field trials. Field trials were performed between June and October 2020. Each field trial had a randomized complete block design with three biological replicates per accession. Each plot had 3 rows, and the length of each plot was 5 m with 0.2 m spacing between each plant, and a spacing of 0.5 m between each row. Each accession was planted in three replicates. Weeds were manually and mechanically removed when needed. 

### 4.3. Data Collection

A total of 10 quantitative traits were scored (Supplementary Table S1), including plant height (PH), nodes number of the main stem (NNS), number of plant branches (NPB), days to flowering (DF), days to maturity (DM), number of pods per plant (NPP), number of seeds per pod (NSP), 100-seed weight (100-SW), seeds weight per plant (SWP), and plot yield (PY). 100-seed weight (100-SW) was recorded from plants were harvested and 100 seeds were selected at random, which measured by an automatic seed-size analyzing system (SC-G, Wanshen, Hangzhou). For each accession, seeds weight per plant (SWP) and plot yield (PY) were obtained using an electronic balance. Days to flowering (DF) was recorded as the number of days to achieve 50% flowering in whole plot. Days to maturity (DM) was recorded as the number of days to achieve the maturity in whole plot. Plant height (PH), nodes number of the main stem (NNS), number of plant branches (NPB), number of pods per plant (NPP), and number of seeds per pod (NSP) were measured after maturity.

### 4.4. Statistical Analysis

All phenotypic traits were analyzed using Excel 2010, and ANOVA analysis and least significant differences (LSD) (*p* ≤ 0.05) were conducted by SPSS 19.0. The min, max, mean, correlation coefficient, and diversity index of each trait were calculated to determine the relationship among traits studied. According to the method of Shannon-Weaver [50] to calculate the diversity index of traits studied. The genotypic coefficient variation (GCV) and phenotypic coefficient variation (PCV) were calculated by the method of Baye [51]. Estimated values of PCV and GCV were categorized as described by Oluwaseyi (between 0% and 10% for low, between 10% and 20% for intermediate, and above 20% for high) [49]. Broad-sense heritability (h^2^_B_) was calculated by the method of Aydin [52] and the expected genetic advance (GA) following the method of Ridzuan [53]. The heritability was classified between 0% and 30% as low, between 30% and 60% as intermediate and high when it was greater than 60% [54]. GA between 0% and 10% as low, between 10% and 20% as intermediate, and greater than 20% as high [55]. Correlation analysis, principal component analysis, and cluster analysis were conducted using the corrplot package [56], princomp function, and hierarchical cluster analysis in R [57].

## 5. Conclusions

In the present study, a total of 475 adzuki bean accessions were evaluated based on 10 agronomic traits and yield characters in two locations, showing a large amount of variation occurred in these evaluated traits. There were significant differences between wild adzuki bean accessions and the other two types of adzuki bean accessions. The NSP, 100-SW, and NPB had a high heritability, indicating that the heritability of these traits is less influenced by the environment. However, the DF, DM, NPP, SWP, and PY were observed as having a low heritability. Some accessions with excellent characters have been screened out which can be used for adzuki bean breeding.

## Figures and Tables

**Table 1:** The phenotype of 10 traits of adzuki beans in two locations.

Traits	Min		Max		Mean ± SD		CV (%)		SHDI	
	Nanning	Nanyang	Nanning	Nanyang	Nanning	Nanyang	Nanning	Nanyang	Nanning	Nanyang
PH	13.5	30.0	130.5	193.9	27.7 ± 10.8 ^b^	69.3 ± 29.3 ^a^	39.0	42.2	1.5	1.3
NNS	6.7	12.0	22.0	27.7	11.6 ± 1.8 ^b^	19.7 ± 2.4 ^a^	15.6	12.3	1.9	1.9
NPB	0	0.3	9.0	10.3	1.3 ± 0.8 ^b^	3.5 ± 1.6 ^a^	59.7	56.2	0.8	1.7
DF	33.7	39.7	62.3	86.3	42.3 ± 4.6 ^b^	56.7 ± 10.4 ^a^	10.9	18.3	1.3	2.0
DM	63.3	74.0	98.0	106.0	73.9 ± 6.5 ^b^	90.1 ± 10.0 ^a^	8.7	11.1	1.3	1.8
NPP	2.3	2.0	80.3	109.7	15.2 ± 9.0 ^b^	20.2 ± 12.8 ^a^	59.3	63.6	1.8	1.8
NSP	1.7	2.0	10.7	11.0	6.6 ± 1.4	6.6 ± 1.4	21.2	20.6	2.0	2.0
100-SW	1.5	1.7	18.5	15.5	8.5 ± 2.9	8.5 ± 2.4	34.7	28.1	1.6	1.8
SWP	1.4	1.3	33.3	35.4	8.0 ± 4.4 ^b^	11.9 ± 6.0 ^a^	54.6	50.4	1.8	1.9
PY	14.7	51.3	1799.6	2312.6	403.6 ± 297.2 ^b^	620.5 ± 359.2 ^a^	73.6	57.9	1.9	1.9

PH, plant height (cm); NNS, nodes number of the main stem; NPB, number of plant branches; DF, days to flowering; DM, days to maturity; NPP, number of pods per plant; NSP, number of seeds per pod; 100-SW, 100-seed weight (g); SWP, seeds weight per plant (g); PY, plot yield (g); CV, coefficient variation; SHDI, Shannon’s Diversity Index. The different lowercase letters (a,b) indicate to be statistical significance at 0.05 level.

**Table 2:** Estimates of variance components for traits studied of adzuki bean accessions.

Source of Variation	df	PH	NNS	NPB	DF	DM	NPP	NSP	100-SW	SWP	PY
Blocks	2	306.85 ***	422.44 ***	38.00 ***	59.63 ***	12.67 ***	377.65 ***	0.00	0.77 ***	539.12 ***	17,837.36 ***
Genotypes (G)	1	1,234,467.94 ***	47,116.40 ***	3317.76 ***	147,787.2 *	187,718.21	17,608.09	1.82 ***	0.03 ***	10458.68	33,519,238.06 **
Environments (E)	474	2214.93 ***	20.38 ***	7.72 ***	208.92 ***	224.31 ***	320.58 ***	10.93	36.06	88.16 ***	362,497.51 ***
G x E	474	704.12 ***	7.02 ***	2.09 ***	178.92 ***	200.42 ***	417.62 ***	0.54 ***	6.87 ***	77.14 ***	289,615.57 ***
Error	1898	2.44	0.11	0.16	0.85	2.19	1.55	0.42	0.00	0.13	4.32

PH, plant height (cm); NNS, nodes number of the main stem; NPB, number of plant branches; DF, days to flowering; DM, days to maturity; NPP, number of pods per plant; NSP, number of seeds per pod; 100-SW, 100-seed weight (g); SWP, seeds weight per plant (g); PY, plot yield (g). *, **, *** indicate significance at *p* < 0.05, *p* < 0.01 and *p* < 0.001, respectively.

**Table 3:** Variance components, heritability, and genetic advance for agronomic and yield traits.

Traits	Mean	V_g_	V_p_	GCV (%)	PCV (%)	RD (%)	h^2^_B_ (%)	GA (%)
PH	48.5	251.8	486.7	33.1	46.0	28.1	51.7	49.1
NNS	15.6	2.2	4.4	9.6	13.5	29.0	50.4	14.0
NPB	2.4	1.0	1.6	58.7	73.5	20.1	63.9	96.7
DF	49.5	3.5	66.1	3.8	16.4	76.8	5.4	1.8
DM	82.0	3.4	78.1	2.3	10.8	79.1	4.4	1.0
NPP	17.7	16.0	126.0	23.8	66.9	64.4	12.7	17.5
NSP	6.6	1.4	1.8	17.8	20.2	11.8	77.8	32.3
100-SW	8.5	4.9	7.2	26.1	31.7	17.5	68.0	44.3
SWP	10.0	2.0	27.5	15.4	57.6	73.3	7.2	8.5
PY	512.1	48,667.7	434,715.4	21.7	65.0	66.5	11.2	15.0

PH, plant height (cm); NNS, nodes number of the main stem; NPB, number of plant branches; DF, days to flowering; DM, days to maturity; NPP, number of pods per plant; NSP, number of seeds per pod; 100-SW, 100-seed weight (g); SWP, seeds weight per plant (g); PY, plot yield (g); V_g_, genotypic variation; V_p_, phenotypic variation; GCV, genotypic coefficient variation; PCV, phenotypic coefficient variation; RD, relative differences; h^2^_B_, broad-sense heritability; GA, genetic advance.

**Table 4:** Trait contributions, eigenvalues, and cumulative percentage of the components.

Traits	PC1	PC2	PC3
PH	−0.47	−0.12	0.05
NNS	−0.29	−0.21	0.23
NPB	−0.41	−0.06	0.19
DF	−0.21	−0.44	−0.23
DM	−0.15	−0.45	−0.31
NPP	0.07	−0.03	0.63
NSP	−0.18	−0.23	−0.33
100-SW	0.40	−0.02	−0.41
SWP	0.24	−0.33	0.43
PY	0.20	−0.32	0.30
Eigen values	3.75	2.94	1.99
Contribution rate (%)	31.26	24.49	16.56
Accumulative contribution rate (%)	31.26	55.75	72.31

PH, plant height (cm); NNS, nodes number of the main stem; NPB, number of plant branches; DF, days to flowering; DM, days to maturity; NPP, number of pods per plant; NSP, number of seeds per pod; 100-SW, 100-seed weight (g); SWP, seeds weight per plant (g); PY, plot yield (g).

**Table 5:** The phenotype of traits of five groups by cluster analysis.

	I	II	III	IV	V
Number of members	143	170	64	74	24
PH	41.7 ± 12.0	48.9 ± 12.2	43.5 ± 7.2	40.0 ± 6.9	114.8 ± 12.5
NNS	15.1 ± 1.8	15.9 ± 1.6	15.7 ± 1.4	15.0 ± 2.0	18.3 ± 2.3
NPB	1.4 ± 0.8	1.6 ± 0.6	1.8 ± 0.6	1.5 ± 0.6	5.7 ± 1.0
DF	47.1 ± 5.6	51.8 ± 5.7	47.8 ± 5.3	49.2 ± 4.3	55.8 ± 2.0
DM	79.9 ± 6.3	84.0 ± 5.7	80.1 ± 5.8	82.8 ± 5.4	86.1 ± 4.6
NPP	15.0 ± 5.1	16.7 ± 6.25	25.6 ± 8.6	14.0 ± 6.3	13.0 ± 9.5
NSP	5.8 ±1.1	7.5 ± 0.9	6.2 ± 1.1	6.0 ± 1.3	7.6 ± 0.5
100-SW	8.0 ± 1.3	8.0 ± 1.4	9.7 ± 1.5	11.8 ± 2.2	2.5 ± 1.2
SWP	7.3 ± 2.6	10.0 ± 2.8	13.7 ± 3.2	9.0 ± 3.3	2.2 ± 1.5
PY	717.8 ±320.2	1214.4 ± 397.7	1451.2 ± 527.1	998.9 ± 371.6	256.2 ± 110.8

PH, plant height (cm); NNS, nodes number of the main stem; NPB, number of plant branches; DF, days to flowering; DM, days to maturity; NPP, number of pods per plant; NSP, number of seeds per pod; 100-SW, 100-seed weight (g); SWP, seeds weight per plant (g); PY, plot yield (g).

## Data Availability

Not applicable.

## Supplementary Materials

The following are available online at https://www.mdpi.com/article/10.3390/plants11091132/s1, Table S1: Geographical distribution of the accessions used in the study (*n* = 475) and phenotypic traits in two locations.
